# The role of tissue and serum carcinoembryonic antigen in stages I to III of colorectal cancer—A retrospective cohort study

**DOI:** 10.1002/cam4.1814

**Published:** 2018-10-09

**Authors:** Guojun Tong, Wei Xu, Guiyang Zhang, Jian Liu, Zhaozheng Zheng, Yan Chen, Pingping Niu, Xuting Xu

**Affiliations:** ^1^ Department of Colorectal Surgery Huzhou Central Hospital Zhejiang China; ^2^ Central Laboratory Huzhou Central Hospital Zhejiang China; ^3^ Pathological Department Huzhou Central Hospital Zhejiang China

**Keywords:** carcinoembryonic antigen, colorectal carcinoma, prognosis

## Abstract

**Purpose:**

Tissue carcinoembryonic antigen (t‐CEA) and serum carcinoembryonic antigen (s‐CEA) expression profiles are the most useful tumor markers for the diagnosis and evaluation of colorectal cancer (CRC) worldwide; however, their roles in CRC progression remain controversial. This study aimed to compare the prognostic values of both s‐CEA and t‐CEA in CRC.

**Methods:**

A total of 517 patients from January 2006 to December 2010 with stages I‐III CRC were retrospectively examined, with 5‐year postoperative follow‐up and death as end‐points. T‐CEA expression, s‐CEA expression, and clinical pathological parameters were inputted into the SPSS 21.0 software. The Kaplan‐Meier method was used to analyze the 5‐year disease‐free survival (DFS) rate of patients in different tumor node metastasis (TNM) stages based on t‐CEA and s‐CEA expression.

**Results:**

Tumor differentiation and the number of positive lymph node harvests were significantly different among the t‐CEA groups (*P* < 0.001, *P* = 0.002); however, clinicopathological features showed no significant difference. The groups with high s‐CEA and t‐CEA expression had a significantly poorer prognosis than those with low s‐CEA (*P* = 0.021) and t‐CEA (*P* < 0.01) expression, respectively. The multivariate analysis demonstrated that t‐CEA was an independent prognostic factor in CRC (*P* < 0.001), but s‐CEA was not (*P* = 0.339). The 5‐year disease‐free survival rates among the t‐CEA groups were significantly different in stages I, II, and III of CRC (*P* = 0.001, *P* < 0.001, *P* < 0.001), whereas in the s‐CEA groups, this difference was observed only in stage III (*P* = 0.014).

**Conclusion:**

This study shows that postoperative t‐CEA expression is an independent factor associated with poorer CRC prognosis and has a higher prognostic value than that of preoperative s‐CEA expression.

## 1 **I**NTRODUCTION

Colorectal cancer (CRC) is the third leading cause of cancer‐related deaths in the United States.^1^, ^2^ CRC shows little symptoms in its early stage, resulting in regional or distant metastasis in most patients at the time of diagnosis, rendering treatment difficult.^3^ Development of CRC occurs progressively, usually spanning 5‐10 years. This extended time frame provides ample opportunities for treatment, especially during the early stage (including the high‐risk stage II).^4^, ^5^, ^6^ A number of independent prognostic factors have been explored and discovered to date, but none of them have been included in conventional treatment.^7^ Several unresolved issues regarding CRC prognosis still exist, but the survival rates in patients with advanced CRC have recently improved because of the advances in diagnostic and surgical procedures.^8^ Currently, the CRC prognosis mainly depends on the stage of the tumor.^9^ Adjuvant treatment is also selected based on the stage of the tumor, and a number of advanced tumors have shown improved prognosis.^10^ The 5‐year survival rates for patients with stage I, II, and III CRC are approximately 93%, 72‐85%, and 44‐83%, respectively.^11^ The meta‐analysis on adjuvant therapy and prognosis remains controversial.^12^

Since its initial discovery in 1965,^13^ carcinoembryonic antigen (CEA) has remained the most thoroughly investigated tumor marker.^14^

Serum carcinoembryonic antigen (CEA) is recommended as a tumor marker in colorectal cancer (CRC) for tumor detecting and monitoring response to therapy.^15^

It is characterized as a member of CD66 cluster of differentiation, and several studies have provided evidence that CEA protein blocks cell differentiation and thus promote tumor progression.^16^, ^17^ Extensive research has been performed to identify CRC‐specific antigens in the blood. However, only two blood‐based biomarkers are available to monitor patients with CRC. CEA, a high molecular weight glycoprotein, is found in embryonic tissue and colorectal malignancies.^18^ The prognostic value of preoperative serum carcinoembryonic antigen (s‐CEA) and postoperative tissue carcinoembryonic antigen (t‐CEA) has been studied, but the prognostic role of increased s‐CEA and t‐CEA expression in patients with CRC remains unknown.^19^ Elevated CEA levels are considered an indicator of poorer prognosis for resectable CRC and are correlated with cancer progression.^20^ Using this marker, the levels of this marker increases with tumor stage;^21^ CEA levels decrease after tumor resection. However, high CEA levels in the blood are not specific for CRC and may be due to other diseases, such as inflammatory bowel disease, liver disease, pancreatitis, and other malignancies. CEA is still the antigen of choice to predict prognosis after CRC diagnosis and to monitor disease progression.^18^ T‐CEA expression is an important tumor marker in CRC, and elevated levels are associated with poorer prognosis. However, the role of t‐CEA expression in CRC progression remains controversial.

This study aimed to analyze the postoperative pathological t‐CEA and preoperative s‐CEA expressions, and their correlation with clinicopathological features; to determine the relationship between s‐CEA and t‐CEA and the 5‐year disease‐free survival rate using single and multiple factors; and to further explore the relationship between independent factors and CRC prognosis.

## 2 MATERIALS AND METHODS

### 2.1 Patients

**Figure 1:**
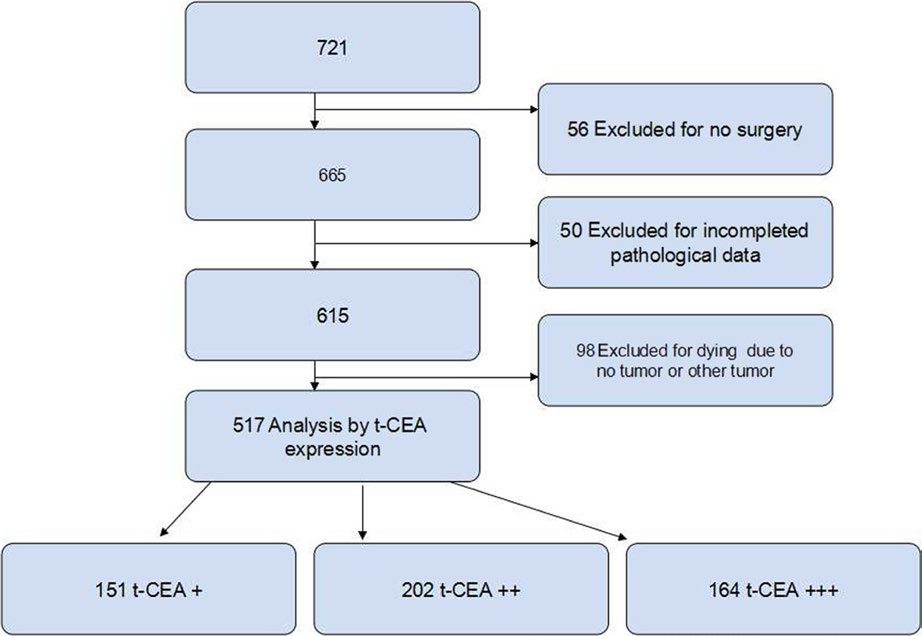
Study flow gram

A total of 721 patients with CRC were admitted to our hospital from January 2006 to December 2010; 204 patients who did not undergo surgery, who had missing postoperative pathologic staging information, and who died due to non‐tumor or other tumor causes were excluded. Finally, 517 patients with stages I‐III CRC were retrospectively examined. The inclusion criteria were as follows: patients diagnosed with CRC through colonoscopy, computed tomography, and pathological tests inside or outside our hospital; no preoperative adjuvant treatment, negative circumcision margin of radical surgery, and normal lymph nodes during dissection; no recurrence such as liver or other organ metastases during the 5‐year follow‐up period and CRC‐related death as a termination event; postoperative routine immunohistochemical analysis and pathological examination; and postoperative chemotherapy determined by the National Comprehensive Cancer Network (NCCN) guidelines. Exclusion criteria were as follows: serious heart, brain, liver, and lung diseases that may affect patient tolerance to the surgery; and non‐CRC factors leading to patient death, pathological interstitial tumors, neuronal tumors, lymphomas, melanomas, and other non‐adenocarcinomas concurrent with CRC (Figure 1). According to the exclusion and inclusion criteria, we try our best to minimize the bias.

### 2.2 Follow‐up

Patients were routinely followed up in the outpatient clinic 2 weeks postoperatively and every 3 months for the first year, then every 6 months for the second year, and every year for the next 3 years till the end of 5 year after operation. Follow‐up data were complemented with phone calls as well as written mails.

### 2.3 Ethics statement

This study was carried out in accordance with the ethical guidelines of the 2008 Declaration of Helsinki and was approved by the ethics committee of Huzhou Central Hospital. Written informed consent was obtained from each patient for the use of serum and tissue samples and medical records for research purposes.

### 2.4 Detection of serum CEA

For each of the selected patients, venous blood was drawn 1 week prior, included s‐CEA tumor in three or ten s‐CEA test is utilized by Shanghai Yu‐ping biotechnology company kit (Shanghai, China), using double antibody one‐step enzyme‐linked immunosorbent assay (ELISA). Experimenters added the sample, standard, and horseradish peroxidase (HRP)‐labeled detection antibody, in that order, to microwells pre‐coated with the CEA capture antibody. After an incubation period, the wells were washed. The 3,3',5,5'‐tetramethylbenzidine substrate was then added; its color first turned blue because of peroxidase catalysis, and then changed to yellow (its final color) because of the acid. The color intensity and human CEA samples were positively correlated. The absorbance (OD value) was measured using a microplate reader at a wavelength of 450 nm to calculate the sample concentration (the normal reference value is 0‐10 ng/L). An s‐CEA level of >10 ng/L is considered high, and ≤10 ng/L is considered low.

### 2.5 T‐CEA immunohistochemistry

Immunohistochemical t‐CEA detection is used as a method to pathologically examine CRC in our hospital. Formalin‐fixed and paraffin‐embedded tumor specimens were cut into 5‐mm‐thick slices, that were then subjected to methyl dewaxing and hydration. The two‐step EnVision immunohistochemistry system was used: the original anti‐CEA antibody (clone No. COL‐1, zm‐0061; Golden Bridge Company, Beijing, China)) was used in a 1:50 dilution, incubated at 4°C; two anti pv8000; finally, an examination under a microscope was performed to determine the percentage of cells positively stained for CEA.

**Figure 2:**
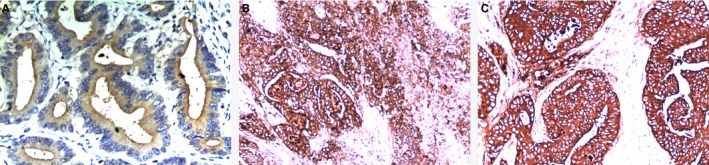
A, B, C: using t‐CEA expression antibody staining, magnification of 200 times, the degree of expression were +, ++, +++, meaning the corresponding proportion (≤25%, >25%, ≤50%, >50% staining) in different degrees. The higher expression of t‐CEA showed the deeper staining.

All slides were independently analyzed by two regularly trained pathologists; the third pathologist was asked to confirm the assessment in case of disagreement. All slides were observed under 100× and 200× magnifications to determine the cell density (+,++, and +++) and the corresponding proportion (≤25%, 25‐50%, and >50%) of stained cells in different regions. From the t‐CEA images shown in Figure 2 (A,B,C), the 200× magnification image was used for better clarity. The 517 patients were divided into three groups based on the different expression levels (+, ++, and +++) of t‐CEA.

### 2.6 Statistical analysis

The SPSS 21.0 (SPSS Inc, Chicago, IL, USA) was used to input all clinical follow‐up data. T‐CEA expression and clinicopathological measurement data in groups with different t‐CEA expression profiles were compared using single factor analysis of variance, whereas counting data was analyzed with Pearson’s *χ* ^2^ test. t‐CEA and s‐CEA values were compared using cross‐table analysis and Pearson’s *χ* ^2^ test. Follow‐up time was defined as the time from operation to death, or from operation to 5 years after operation; CRC‐related death and the 5‐year follow‐up were used as the end‐points (2015.1). The log‐rank test was used to analyze CRC prognoses according to s‐CEA and t‐CEA expression. The Kaplan‐Meier method was used to analyze the 5‐year disease‐free survival rates and hazard ratios (HR). The survival rate was also analyzed using Cox’s regression for univariate and multivariate analysis based on different clinical, pathological, and biochemical variables. The relationship between independent factors related to survival rate and all variables in the first index (input) were carried out. A *P*‐value of <0.05 (double test) was considered statistically significant.

## 3 RESULTS

### 3.1 General data

A total of 517 patients with CRC (261 [50.5%] men and 256 [49.5%] women) were included in this study. Patients were classified based on t‐CEA expression (from low to high: +, ++, +++); the complete cohort included 77 (29.5%), 90 (34.5%), and 94 (36%) male patients, and 74 (28.9%), 112 (43.8%), and 70 (27.3%) female patients, respectively. The median age was 66 (range, 17‐89) years.

### 3.2 Clinicopathological features of patients with different t‐CEA expression profiles

T‐CEA expression was analyzed and categorized based on gender, age, ASA classification, location, operation, invasion depth, differentiation, tumor node metastasis (TNM) classification, number of lymph harvests, number of positive lymph harvests, and administration of chemotherapy as clinicopathological features. Pearson’s *χ* ^2^ test revealed no significant difference (*P* = 0.052) in these characteristics. The mean age (± standard deviation) of 517 patients was 61.8 ± 13.8, 61.6 ± 15.7, and 62.5 ± 14.5 years, in the groups based on t‐CEA expression (+, ++, +++), respectively. No significant difference was observed when the variance test was applied (*P* = 0.836). Regarding the ASA classification, in the three t‐CEA expression groups (+, ++, +++), 371 patients had stage I CRC [116 (76.8%), 144 (71.3%), and 111 (67.7%)]; 133 patients had stage II CRC [31 (20.5%), 53 (26.2%), and 49 (29.9%)]; and 13 patients had stage III [4 (2.6%), 5 (2.5%), and 4 (2.4)], respectively; no significant differences were observed (*P* = 0.456). The three groups of t‐CEA expression showed no significant difference in terms of tumor location (*P* = 0.127). Operation style, invasion depth, and TNM classification did not also show significant difference in the three t‐CEA expression groups (*P* = 0.138, *P* = 0.42, *P* = 0.101, respectively). The mean (± standard deviation) of tumor size was 3.85 ± 0.98, 3.82 ± 0.86, and 3.93 ± 0.81 in the three t‐CEA groups (+, ++, +++), respectively, but no significant difference was observed after the variance test was applied (*P* = 0.487). The number of lymph node harvests was 14.3 ± 1.9, 14.1 ± 1.7, and 14.2 ± 1.9, respectively, in the three t‐CEA groups (+, ++, +++), with no significant difference after statistical analysis (*P* = 0.661). In contrast, the number of positive lymph node harvests was 1.37 ± 1.4, 1.52 ± 1.6, and 2.01 ± 2.0, respectively, in the three t‐CEA groups (+, ++, +++), with a statistically significant increase with an increase in t‐CEA expression (*P* = 0.002). Among the 517 patients, 449 underwent chemotherapy [124 (82.1%), 182 (90.1%), and 147 (89.6%) in the three t‐CEA expression groups, (+, ++, +++), respectively], and 68 patients did not accept chemotherapy [12 (17.9%), 20 (9.9%), and 17 (10.4%) in the t‐CEA expression groups, (+, ++, +++), respectively]. However, the results showed no significant difference (*P* = 0.739; Table 1).

**Table 1:** Clinicopathological features of CEA in different tissues n (%); Mean number of $<![CDATA\left[\overset{x}{\pm }s\right]]>$

	N	T‐CEA+ 151	T‐CEA++ 202	T‐CEA+++ 164	*P* value
Gender					
Male	261	77 (51)	90 (44.6)	94 (57.3)	0.052
Female	256	74 (49)	112 (55.4)	70 (42.7)	
Age	517	61.8 ± 13.8	61.6 ± 15.7	62.5 ± 14.5	0.836
ASA classification					
I	371	116 (76.8)	144 (71.3)	111 (67.7)	0.456
II	133	31 (20.5)	53 (26.2)	49 (29.9)	
III	13	4 (2.6)	5 (2.5)	4 (2.4)	
Location					
Ileocecum	32	9 (6)	6 (3)	17 (10.4)	0.127
Right colon	41	10 (6.6)	21 (10.4)	10 (61)	
Transverse colon	86	27 (17.9)	34 (16.8)	25 (15.2)	
Left colon	112	36 (13.8)	38 (18.8)	38 (23.2)	
Sigmoid colon	52	13 (8.6)	19 (9.4)	20 (12.2)	
Rectum	194	56 (37.1)	84 (41.6)	54 (32.9)	
Operation method					
RHC	93	24 (15.9)	34 (16.8)	35 (21.3)	0.138
LHC	223	69 (45.2)	82 (40.6)	72 (43.9)	
HO	9	2 (1.3)	4 (2)	3 (1.8)	
AR	143	45 (29.8)	66 (32.7)	32 (19.5)	
APR	49	11 (7.3)	16 (7.9)	22 (13.4)	
Invasive depth					
T1	48	15 (9.9)	22 (10.9)	11 (6.7)	0.42
T2	86	23 (15.2)	37 (18.3)	26 (15.9)	
T3	254	68 (45)	101 (50)	85 (51.8)	
T4	129	45 (29.8)	42 (20.8)	42 (25.6)	
Differentiation					
Well	97	26 (17.2)	45 (22.3)	26 (15.9)	<0.001*
Moderate	306	115 (76.2)	123 (60.9)	68 (41.5)	
Poorly or undifferentiation	114	10 (6.6)	34 (16.8)	70 (42.7)	
Tumor Size (cm)	517	3.85 ± 0.98	3.82 ± 0.86	3.93 ± 0.81	0.487
TNM classification					
I	67	27 (17.9)	27 (13.4)	13 (7.9)	0.101
II	138	35 (23.2)	53 (26.2)	50 (30.5)	
III	312	89 (58.9)	122 (60.4)	101 (61.6)	
NO. of lymph harvest	517	14.3 ± 1.9	14.1 ± 1.7	14.2 ± 1.9	0.661
Positive NO. of lymph harvest	517	1.37 ± 1.4	1.52 ± 1.6	2.01 ± 2.0	0.002*
Chemotherapy					
Yes	449	124 (82.1)	182 (90.1)	147 (89.6)	0.739
No	68	12 (17.9)	20 (9.9)	17 (10.4)	

* *P* < 0.05, statistical difference.

### 3.3 Relationship between t‐CEA and s‐CEA

The proportions of preoperative low and high t‐CEA expression were 39.8% (206/517) and 60.2% (311/517), respectively, and those of postoperative t‐CEA expression (+, ++, +++) were 29.2% (151/517), 39.1% (202/517), and 31.7% (164/517), respectively. There was no significant relationship between t‐CEA and s‐CEA expression (*χ* ^2^ = 1.146, *P* = 0.564; Table 2). Pearson’s correlations analysis showed that the correlation between the two is 0.016, which is far from 1 and *P* = 0.715.

**Table 2:** Relationship between preoperative serum CEA (s‐cea) level and tumor tissue CEA (t‐cea)

	Tumor tissue T‐CEA expression			*χ* ^2^	*P*
	T‐CEA+ (n = 151)	T‐CEA++ (n = 202)	T‐CEA+++ (n = 164)		
Serum S‐CEA					
≤10 ng/mL (n = 206)	59	86	61	1.146	0.564
>10 ng/mL (n = 311)	92	116	103		

### 3.4 Univariate analysis of immunohistochemical and survival rate in clinical pathology

Gender, tumor location, invasion depth, differentiation, TNM, s‐CEA, t‐CEA, and chemotherapy were variables included in the univariate survival analysis. The average survival time for men was 53.6 months, with a cumulative 5‐year disease‐free survival rate of 60.1% (95% confidence interval [CI]: 51.76‐54.35), whereas that for women was 54.77 months, with the 5‐year cumulative survival rate being 69.2% (95% CI: 53.46‐56.08); these rates were significantly different (*P* = 0.022). Ileocecum cancer had a mean survival time of 51.28 months, with a 52.9% 5‐year survival rate (95% CI: 47.25‐55.31); right colon cancer, 53.71 months with a 73.2% 5‐year survival rate (95% CI: 50.35‐57.06); transverse colon cancer, 53.86 months with a 61.4% 5‐year survival rate; left colon cancer, 53.67 months with a 64.8% 5‐year survival rate (95% CI: 51.62‐55.73); sigmoid colon cancer, 55.20 months with a 63.2% 5‐year survival rate (95% CI: 52.99‐57.41); and rectum cancer, 54.19 months with a 66.4% 5‐year survival rate (95% CI: 52.66‐55.73). However, no significant differences were observed among patients with different tumor locations (*P* = 0.616). In the low s‐CEA group, the average survival time was 55.01 months, with a 70.5% survival rate (95% CI: 53.66‐56.36), whereas that in the high s‐CEA group was 53.17 months, with a 60.6% survival rate (95% CI: 51.93‐54.41); the difference was statistically significant (*P* = 0.021). Patients with t‐CEA expression “+” had an average survival time of 58.35 months, with an 88.6% survival rate (95% CI: 57.46‐59.25); those with t‐CEA expression “++,” 55.15 months with a 70.3% survival rate (95% CI: 53.78‐56.51); and those with t‐CEA expression “+++,” 48.28 months with a 35.5% survival rate (95% CI: 46.35‐50.22); differences among the three groups were statistically significant (*P* < 0.01). Other variables, such as invasion depth, differentiation, TNM, and chemotherapy, were also significantly different among the groups (*P* = 0.022, *P* < 0.01, *P* < 0.01, *P* < 0.01, respectively). The details of average survival times, 5‐year cumulative survival rates, Hazard Ratio (HR) for risk, and 95% CI for survival time are shown in Table 3.

**Table 3:** Univariate analysis of prognosis of colorectal cancer

Factor	Mean survival time (mo, Follow‐up 60 mo)	Hazard Ratio (HR)	95% CI for survival time	5 y survival rate (%)	*P* value
Gender					
Male	53.06	1	51.76‐54.35	60.1	0.022*
Female	54.77	0.713	53.46‐56.08	69.2	
Location					
Ileocecum	51.28	1	47.25‐55.31	52.9	0.616
Right colon	53.71	0.533	50.35‐57.06	73.2	
Transverse colon	53.86	0.747	51.64‐56.07	61.4	
Left colon	53.67	0.681	51.62‐55.73	64.8	
Sigmoid colon	55.20	0.684	52.99‐57.41	63.2	
Rectum	54.19	0.636	52.66‐55.73	66.4	
Invasive Depth					
T1	58.02	1	56.20‐59.84	85.4	0.022*
T2	54.71	2.805	52.73‐56.70	63.3	
T3	53.45	2.976	52.08‐54.81	62.8	
T4	52.72	3.220	50.73‐54.72	61.0	
Differentiation					
Well	59.59	1	58.87‐60.03	95.8	<0.01*
Moderate	56.52	6.933	55.65‐57.39	74.4	
Poorly, undifferentiation	42.05	49.911	39.71‐44.38	53.1	
TNM					
Ⅰ	59.81	1	59.59‐60.03	95.3	<0.01*
II	56.95	5.764	55.70‐58.20	75.8	
III	51.29	13.877	49.95‐52.64	53.1	
Serum CEA					
≤10 ng/mL	55.01	1	53.66‐56.36	70.5	0.021*
>10 ng/mL	53.17	1.433	51.93‐54.41	60.6	
Tumor tissue CEA					
+	58.35	1	57.46‐59.25	88.6	<0.01*
++	55.15	2.895	53.78‐56.51	70.3	
+++	48.28	8.381	46.35‐50.22	35.5	
Chemotherapy					
Yes	53.06	1	52.02‐54.09	60.1	<0.01*
No	59.50	0.121	58.83‐60.15	93.9	

* *P* < 0.05 statistical difference.

### 3.5 The 5‐year disease‐free survival (DFS) rate of t‐CEA and s‐CEA in different TNM stages

**Figure 3:**
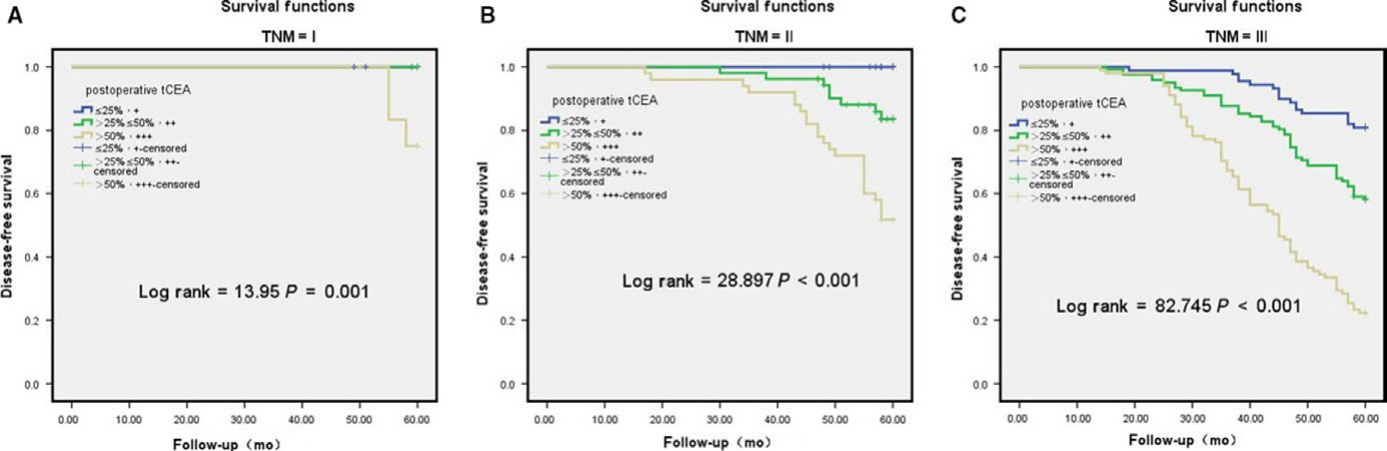
A, B, C: T‐CEA group were calculated for different TNM staging by 5‐y disease‐free survival (DFS) by univariate analysis. A: I, B: II, C: III. I, II, III stage were significantly different, *P* < 0.05

In the t‐CEA + group, the 5‐year survival rates were 100% (n = 27), 100% (n = 35), and 80.9% (n = 89) for patients with stage I, II, and III CRC, respectively; in the t‐CEA ++ group, 100% (n = 27), 83.6% (n = 53), and 58.2% (n = 122), respectively; and in the t‐CEA +++ group, 75% (n = 13), 51.8% (n = 50), and 22.4% (n = 101), respectively. The log‐rank test revealed that the survival rates were significantly different among the t‐CEA expression groups (*χ* ^2^ = 13.95, *P* = 0.001; *χ* ^2^ = 28.9, *P* < 0.001; *χ* ^2^ = 82.7, *P* < 0.01; Figure 3A,B,C).

**Figure 4:**
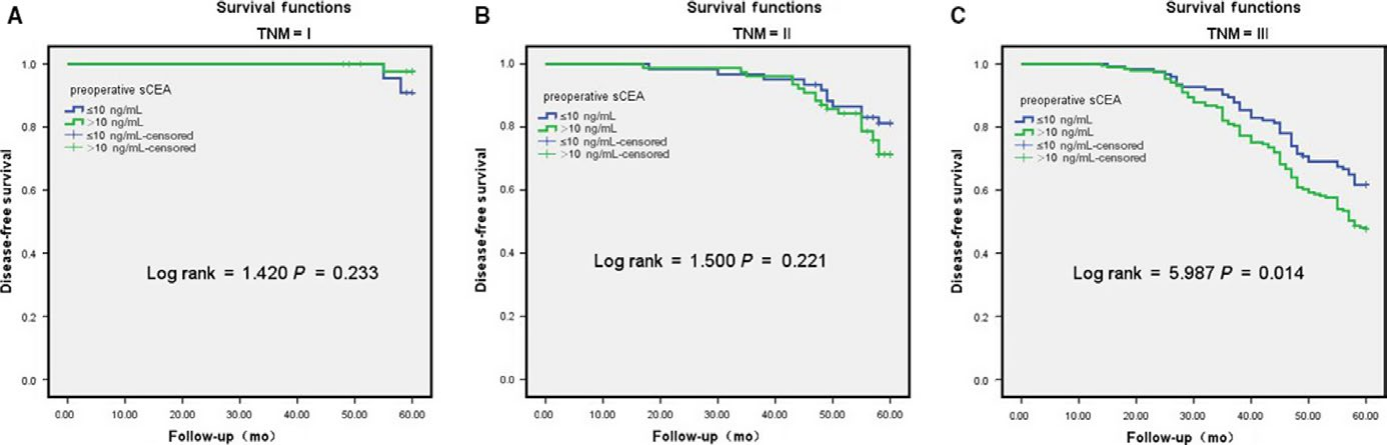
A, B, C: S‐CEA group were calculated for different TNM staging by 5‐y disease‐free survival (DFS) by univariate analysis. A: I, B: II, C: III. Only III stage were significantly different, *P* < 0.05, and I, II stage was not significant, *P* > 0.05

In the low s‐CEA group, the 5‐year survival rates were 90.9% (n = 22), 81.2% (n = 61), and 61.7 (n = 123) in individuals with stage I, II, and III CRC, respectively, whereas those in the high s‐CEA group were 97.6% (n = 45), 71.4% (n = 77), and 47.6 (n = 189), respectively. The log‐rank test revealed no significant difference in stages I and II (*χ* ^2^ = 1.42, *P* = 0.233, *χ* ^2^ = 1.50, *P* = 0.221), but a statistical difference was observed in stage III (*χ* ^2^ = 5.987, *P* = 0.014; Figure 4A,B,C).

### 3.6 **Multivariate analysis on the prognostic factors of **CRC

**Figure 5:**
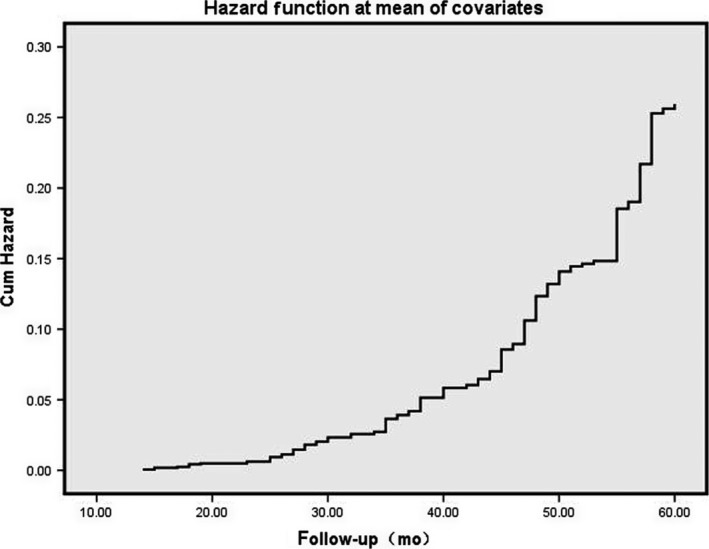
The indicators with significant significance in univariate analysis were included in the COX risk regression analysis, using the entry method, and the first indicator was used as the indicator parameter to obtain the risk pattern. Multivariate COX regression analysis of risk maps suggest that t‐CEA is an independent prognostic factor and s‐CEA is not

To determine the independent predictors of CRC prognosis among the clinical, pathological, and biochemical indices, we used Cox proportional hazard model analysis. Variables such as gender, tumor invasion depth, degree of differentiation, TNM stage, s‐CEA, t‐CEA, and chemotherapy which showed statistical difference during the univariate analysis were included in this model. Regarding gender, the hazard ratio (HR) (95% CI) for women vs. men was 1.128 (1.533‐0.0.830), which was not significant (*P* = 0.442). Regarding invasion depth, the HR (95% CI) for T2 vs. T1, T3 vs. T1, and T4 vs. T1 were 0.299 (0.704‐0.127), 1.013 (1.631‐0.630), and 0.861(1.226‐0.605), respectively, showing statistical significance (*P* = 0.039). Regarding the degree of differentiation, the HR (95% CI) for moderate vs. well, poor or undifferentiated vs. well were 0.035 (0.098‐0.012), 0.231 (0.325‐0.165), respectively, showing statistical significance (*P* < 0.001). Regarding TNM, HR (95% CI) for stage II vs. I and III vs. I were 0.24 (0.265‐0.002) and 0.609 (0.918‐0.404), respectively, showing statistical significance (*P* = 0.001). Regarding s‐CEA, the HR (95% CI) for high vs. normal was 0.856 (1.178‐0.622), showing no statistical significance (*P* = 0.339). Regarding t‐CEA expression, HR (95% CI) for expressions “++” vs “+” and “+++” vs “+” were 0.177 (0.308‐0.102) and 0.523 (0.739‐0.371), respectively, showing statistical significance (*P* < 0.001). Regarding chemotherapy, the HR (95% CI) for no chemotherapy vs chemotherapy treatment was 0.137 (1.141‐0.016), showing statistical significance (*P* = 0.066; Table 4 and Figure 5).

**Table 4:** Multivariate analysis of prognosis of colorectal cancer

Factor	Hazard rate (95% CI)	*P* value
Gender		
F/M	1.128 (1.533‐0.830)	0.442
Invasive depth		
T2/T1	0.299 (0.704‐0.127)	0.039*
T3/T1	1.013 (1.631‐0.630)	
T4/T1	0.861 (1.226‐0.605)	
Differentiation		
Moderate/well	0.035 (0.098‐0.012)	<0.001*
Poor or undifferentiation/well	0.231 (0.325‐0.165)	
TNM stage		
II/Ⅰ	0.24 (0.265‐0.002)	0.001*
III/Ⅰ	0.609 (0.918‐0.404)	
Serum CEA		
High/Normal	0.856 (1.178‐0.622)	0.339
Tissue CEA		
++/+	0.177 (0.308‐0.102)	<0.001*
+++/+	0.523 (0.739‐0.371)	
Chemotherapy		
No/Yes	0.137 (1.141‐0.016)	0.066

* *P* < 0.05statistical difference.

## 4 DISCUSSION

Gold and Freedman^13^, ^22^ discovered CEA in 1965, when they observed it in fetal colon and colon cancers; however, CEA was not observed in the colons of healthy adults and was therefore called a carcinoembryonic antigen. Subsequent studies suggested that CEA was also found in healthy tissues, although its concentration in tumor tissues was 60 times higher than that in non‐tumor tissues.^23^ The CEA gene was recently classified as an immunoglobulin gene super family member.^24^ One recent research showed CEA was a glycoprotein associated with colorectal cancer (CRC) and the changing CEA glycosylation patterns and their role in the development of CRC highlight the importance of glycan variants on CEA for early clinical detection and staging of CRC.^25^

CEA is a widely used tumor marker globally and can not only be detected in the blood of CRC patients but also in the tumor tissue.^26^ The CEA gene and antibody expressions in tumor tissues have already been studied; however, studies on s‐CEA and its prognostic value in CRC, locally and internationally, are relatively limited and are not comprehensive. Therefore, the results remain controversial and unclear.

CEA levels in CRC are known to be associated with preoperative tumor extent, tumor outcomes, and recurrence.^27^ However, so far, no consensus has been achieved regarding its role in tumor responses to chemotherapy, although some authors have investigated the efficacy of CEA monitoring for the evaluation of tumor responses in palliative chemotherapy.^28^, ^29^, ^30^, ^31^, ^32^ Previous studies showed well‐differentiated CRCs produced more CEA in serum and primary tissues than poorly differentiated specimens,^33^, ^34^ it maybe because it was not defined whether free CEA protein or cellular CEA or both take effects in cell differentiation.^35^The relationship between CEA expression and tumors remains unknown. In this study, t‐CEA expression was not significantly associated with tumor location, size, and TNM stage; however, it was significantly correlated with the degree of tumor differentiation and the number of positive lymph node harvests showing that the poorer the degree of tumor differentiation accompanied the higher the incidence of lymph node metastasis and the higher the CEA expression. T‐CEA expression increases with an increasing number of positive lymph node harvests and poorer CRC differentiation. In colon cancer, CEA appears to have various cellular functions, including adhesion, in both intracellular and CEA‐matrix interactions;^36^, ^37^, ^38^, ^39^ signal transduction; and cellular migration,^40^, ^41^, ^42^ suggesting that CEA facilitates tumor invasion and metastasis. Therefore, we can speculate that CEA also plays a similar role in CRC. However, our results demonstrated that t‐CEA was not significantly correlated with the depth and size of tumor invasion.

The American Joint Cancer Commission/tumor node metastasis (AJCC/TNM) classification is widely used as a guideline for staging and represents the best prognostic indicator of outcomes in patients with CRC.^43^, ^44^ Histopathological types are also reported to predict the outcomes in patients with CRC.^45^ In clinical practice, accurate AJCC/TNM staging and histopathological analysis depend on postoperative pathological examination. Recent studies have focused on the prognostic value of serum tumor markers in CRC.^46^, ^47^ Jingtao Wang et al^48^ recommended CEA for prognostic surveillance following curative resection and monitoring of therapeutic responses in advanced diseases. Recently, Kwan Mo Yang et al^49^ found that persistently elevated postoperative s‐CEA expression was significantly correlated with higher recurrence and poorer survival rates in patients with CRC. In their study, 25.6% (318 of 1242) of patients with stages I‐III CRC with abnormal preoperative s‐CEA expression, elevated s‐CEA expression was sustained postoperatively, and those with high pre‐ and postoperative s‐CEA expression exhibited poorer outcomes. The s‐CEA and t‐CEA expression profiles in 517 patients in this study were analyzed based on the CRC prognosis from stages I to III. The results showed that s‐CEA expression was significantly associated with poorer prognosis only in stage III CRC, which was consistent with some previous studies,^50^, ^51^, ^52^ whereas t‐CEA expression was significantly associated with CRC prognosis in stages I to III. Our study illustrated the relationship between s‐CEA and t‐CEA showing there were no relationship and relevance between them. We could think the s‐CEA results and t‐CEA expression were asymmetric.

Some previous studies^53^, ^54^ demonstrated that s‐CEA was an independent prognostic factor for CRC, which was inconsistent with the results of our study We found s‐CEA was only a prognostic factor for CRC, not an independent prognostic factor; however, t‐CEA was not only a prognostic factor but also an independent prognostic factor of CRC by univariate analysis and multivariate analysis. While there were some limitations in this study, such as nonlarge data research, nonprospective study, and no analysis of overall survival (OS) because of some missing follow‐up data.

## 5 CONCLUSION

Preoperative s‐CEA expression is associated with poor outcomes only during stage III CRC and is not an independent prognostic factor, whereas elevated t‐CEA expression is associated with poorer prognosis for stages I‐III of CRC by univariate analysis and multivariate analysis and is an independent prognostic factor. Thus, t‐CEA expression has a higher prognostic value than that of preoperative s‐CEA, with the defection of no repeated detection.

## CONFLICTS OF INTEREST

The authors indicated no potential conflicts of interests.
